# A path to next-generation reproducibility in cheminformatics

**DOI:** 10.1186/s13321-019-0385-0

**Published:** 2019-10-14

**Authors:** Robert D. Clark

**Affiliations:** Simulations Plus, Inc, 42505 10th Street West, Lancaster, CA 93534 USA

**Keywords:** Reproducibility, Consilience, Triangulation, Replicability, Reimplementation, Algorithm validation

## Abstract

Currently, the submission guidelines for the Journal of Cheminformatics say it will “only publish research or software that is entirely reproducible by third parties.” They go on to specify that being reproducible means that anything essential to the conclusion of the paper be freely accessible and states that source code must be provided. I submit that this definition of reproducibility is too narrow—that a cheminformatics method can only truly be replicated by reimplementing it from a detailed, step-by-step high-level description to determine how reliably the algorithm per se does what it is intended to do.

## Introduction

What follows reflects one person’s views on how issues of reproducibility apply to modeling and the cheminformatics support system upon which it depends. One hopes it comes across more as an exhortation to sustain disciplined virtue than as a grumpy polemic raging against the winds of change now blowing through the machine learning community.

## Commentary

The difficulty of reproducing published experimental results is not a new phenomenon, but it became a very visible one after researchers at Bayer and Amgen reported distressingly high failure rates when trying to reproduce literature reports on drug target identification [1] and from preclinical cancer research [2], respectively. Many factors contribute to such irreproducibility, but two biases—publication and confirmation bias—are particularly problematic. Failure to properly distinguish association from causation exacerbates the psychological effects of both: not publishing cases where no association is found makes reported associations seem more significant than they actually are, as does publishing an inadequately supported conclusion that people expect or would like to be true.

My own background is in analytical biochemistry, which has shaped my perspective on what reproducibility means. There, the point of rerunning a biological experiment is to reassure oneself that others will be able to do it and get *similar* results, not that you or someone else will get *exactly* the same numbers. Indeed, identical or unduly precise (e.g., “1.0000”) biochemical measurements typically prompt a check for a duplicated entry or a missing qualifier like “ > ”, a habit that still pays dividends in data curation. The same is true for organic synthesis: exactly reproducing a yield or a melting point is more likely to be an error or a coincidence than an assurance that the experiment was truly replicated.

At the end of the day, conclusions need to be both specifically *and* generally correct. If your work is going to be useful to others, they need to know how accurately and how precisely they will be able to reproduce your particular result, but also how confidently they can apply the tools you describe in analogous situations. Reproducing published analytical or synthesis work in this sense is best seen as a form of consilience or triangulation, in which confidence in a conclusion increases because it has been reached from multiple directions, fortuitously or by design [3].

“Validation” represents something different in computational disciplines like cheminformatics because there is no explicit replication of a method or prediction: absent bugs, a computer program will produce the same output every time all inputs are the same and *all inputs can be controlled*.^1^ That fact is difficult to reconcile with the current identification of cheminformatic “reproducibility” as being adequately satisfied by publication of source code and all attendant data. Absent outright fraud, simply rerunning an analysis provides no indication whether a conclusion or prediction is correct or not, nor—perhaps more importantly—whether it is correct for the wrong reason [4]. Many systematic errors can be found by directly examining the code or the data—in principle. Unfortunately, reimplementing a complicated algorithm is often difficult and frustrating, especially when one’s own output doesn’t match that for the published program. It is tempting to simply accept the validity of the program or the data or both unconditionally, but doing so is a recipe for propagation of errors. Then, too, direct inspection of the code risks falling prey to confirmation bias: code that looks right line by line and routine by routine may still not be doing what it is supposed to be doing.

We as a community can address this by broadening our understanding of what “reproducing” a cheminformatics study means. Advances in methodology are best evaluated by independent reimplementation of the algorithm as described in detail by the original authors in step-by-step text or pseudocode [5–7]. The Journal should support such endeavors, particularly where an original publication is not completely transparent because of commercial or proprietary considerations, especially where neither source code nor scripts were part of the original report. My own experience with reimplementation is that it almost always clarifies ambiguities in the original publication and sometimes identifies mistakes in the original code, just as good refactoring does.

It is important that any such proof-of-principle reimplementation focus on producing simple and interpretable code, maximizing clarity and interpretability while minimizing the risk of introducing secondary errors. If possible, the method’s originators should participate in the process: besides being appropriate as a matter of professional etiquette, such participation will minimize the amount of time and effort wasted due to misunderstandings or pilot error. Everyone’s software is likely to be improved or clarified as a result, and the field will move forward.

A reimplementation study should go beyond (more or less) reproducing the original published results. In particular, it should also apply the method to a fresh test set; if that is not feasible, the original input test data should be perturbed somehow—e.g., by renumbering atoms [8] or modifying parameters [9]. Doing so will go a long way towards mitigating potential publication bias: no matter how carefully test sets are chosen, variations with better looking test set statistics are more likely to be reported than are those that those that perform less well on that particular data set. Published performance statistics are necessarily overly optimistic to a greater or lesser degree as a result.^2^

One hopes that we will eventually come to see reimplementing a method as the sincerest form of validation, especially when the original publication is not fully transparent for historical reasons or because critical information is proprietary. For it to work, however, we need to require that authors thoroughly explain what they intend their program to do and to provide results for representative data. This is best done through good, clear writing and illustrations—pseudocode or its equivalent, tables and molecular structures.

Providing source code is valuable in its own right but is not a substitute for reimplementation, especially when the code is complicated by the interface management, external dependencies and pilot-error checking characteristic of industrial-grade applications as well as extraneous verbiage [10]. The parallel in analytical biochemistry is sharing reagents. Being provided with an antibody or cell line facilitates reproducibility in the narrow sense, but checking that the shared reagent is fit for the intended use is still critical to reproducibility in the broader sense [11, 12]. Reimplementing a program is the closest we can come to doing that in cheminformatics.

## Conclusion

Conscientiously reimplementing programs as described here will help the community begin to address the more substantive reproducibility issues in cheminformatics that lie beyond the comparatively trivial ones we address now. Absent a sufficiently precise and detailed description of what a program is intended to do, it cannot be fully replicated, and until such replication is done, any conclusion based on a program is just an hypothesis that hopes to become a theory someday.

## Data Availability

Not applicable.

## Footnotes

1 Random perturbations can be introduced by seeding a random number generator on the fly from some arbitrary input (e.g., the clock), but failing to record the seed used makes it nearly impossible to fully debug a program and will aggravate most users.

2 Most conscientious scientists report at least a few of the approaches that were tried but failed to work, or report results for a handful of parameter combinations and then pick the best, a practice that carries its own kind of bias. Exhaustive reporting of everything tried that didn’t work would constitute a cure worse than the disease; it would be too much information.
